# Local leptin production in osteoarthritis subchondral osteoblasts may be responsible for their abnormal phenotypic expression

**DOI:** 10.1186/ar2925

**Published:** 2010-02-08

**Authors:** Marie-Solange Mutabaruka, Mohamed Aoulad Aissa, Aline Delalandre, Martin Lavigne, Daniel Lajeunesse

**Affiliations:** 1 Unité de recherche en Arthose, Centre de recherche du Centre Hospitalier de l'Université de Montréal (CR-CHUM), Hôpital Notre-Dame, 1560 rue Sherbrooke Est, Montréal, QC H2L 4 M1, Canada; 2 Chirurgie Orthopédique, Hôpital Maisonneuve-Rosemont, 5415 boul. l'Assomption, Montréal, QC H1T 2 M4, Canada

## Abstract

**Introduction:**

Leptin is a peptide hormone with a role in bone metabolism and rheumatic diseases. The subchondral bone tissue plays a prominent role in the pathophysiology of osteoarthritis (OA), related to abnormal osteoblast (Ob) differentiation. Although leptin promotes the differentiation of Ob under normal conditions, a role for leptin in OA Ob has not been demonstrated. Here we determined if endogenous leptin produced by OA Ob could be responsible for the expression of the abnormal phenotypic biomarkers observed in OA Ob.

**Methods:**

We prepared primary normal and OA Ob from subchondral bone of tibial plateaus removed for knee surgery of OA patients or at autopsy. We determined the production of leptin and of the long, biologically active, leptin receptors (OB-Rb) using reverse transcriptase-polymerase chain reaction, ELISA and Western blot analysis. We determined the effect of leptin on cell proliferation by BrdU incorporation and 3-(4,5-Dimethylthiazol-2-yl)-2,5-diphenyltetrazolium bromide (MTT) assays, and we determined by Western blot analysis phospho 42/44 MAPK (p42/44 Erk1/2) and phospho p38 levels. We then determined the effect of the addition of exogenous leptin, leptin receptor antagonists, inhibitors of leptin signaling or siRNA techniques on the phenotypic features of OA Ob. Phenotypic features of Ob were determined by measuring alkaline phosphatase activity (ALP), osteocalcin release (OC), collagen type 1 production (CICP) and of Transforming Growth Factor-β1 (TGF-β1).

**Results:**

Leptin expression was increased approximately five-fold and protein levels approximately two-fold in OA Ob compared to normal. Leptin stimulated its own expression and the expression of OB-Rb in OA Ob. Leptin dose-dependently stimulated cell proliferation of OA Ob and also increased phosphorylated p42/44 Erk1/2 and p38 levels. Inactivating antibodies against leptin reduced ALP, OC, CICP and TGF-β1 levels in OA Ob. Tyrphostin (AG490) and piceatannol (Pce), inhibitors of leptin signaling, reproduced this effect. Inhibition of endogenous leptin levels using siRNA for leptin or inhibiting leptin signaling using siRNA for OB-Rb expression both reduced ALP and OC about 60%. Exogenous leptin addition stimulated ALP, yet this failed to further increase OC or CICP.

**Conclusions:**

These results suggest that abnormal production of leptin by OA Ob could be responsible, in part, for the elevated levels of ALP, OC, collagen type 1 and TGF-β1 observed in these cells compared to normal. Leptin also stimulated cell proliferation, and Erk 1/2 and p38 signaling. Taken together, these data suggest leptin could contribute to abnormal osteoblast function in OA.

## Introduction

Osteoarthritis is characterized by progressive articular cartilage loss, appositional new bone formation and sclerosis of the subchondral trabeculae and growth plate, formation of osteophytes, and an imbalance between loss of cartilage, due to matrix degradation, and an attempt to repair this matrix [1,2]. Synovitis is often observed and is considered to be secondary to the changes in hard tissues within the joint. Despite major progress in the last few years, we still have a lot to learn about the etiology, pathogenesis and progression of this disease [3]. The slowly progressive and multifactorial nature of the disease, its cyclical course, where a period of active disease is followed by a period of remission, have limited our comprehension of OA. Risks factors for this disease in humans include age, gender, genetic predisposition, mechanical stress and/or joint trauma, and obesity [3,4].

A relationship exists between obesity/fat mass and bone mass, while the mechanisms responsible for this are still not fully understood, and OA patients have a better preserved bone mass [5,6], independently of body weight [7], than healthy individuals. High body mass index (BMI) and increased bone mineral density (BMD) suggest new bone synthesis exceeds degradation in OA. In support of this hypothesis, osteocalcin (a marker of bone formation) in synovial fluid and serum osteopontin (a bone specific matrix protein) were significantly higher in patients with knee scan abnormalities [8]. Gevers and Dequeker showed elevated serum osteocalcin levels in women with hand osteoarthritis, and elevated osteocalcin in cortical bone explants [9]. This group also reported that IGF-I and II, and TGF-β levels are higher in samples of iliac crest bone of patients with OA [10], at a site distant from weight bearing joints, suggesting a generalized bone metabolic dysfunction. Our group showed that *in vitro *OA Ob produced higher IGF-1 and TGF-β levels compared to normal [11,12].

Leptin, the product of the obese (*ob*) gene, is a 16-kDa secreted protein that is produced by white adipocytes and placenta, and functions as an afferent signal to influence energy homeostasis through effects on energy intake and expenditure [13-15]. When leptin is mutated it results in obesity in the ob/ob mouse [13]. It is now evident that leptin is also expressed in osteoblasts [16]. Moreover, in addition to its effects on the central nervous system (CNS), leptin acts through high affinity leptin receptors on cells in peripheral tissues [17-19]. Leptin suppresses specific biochemical processes contributing to lipid accumulation and adipocyte differentiation [20]. The long, signaling-competent isoform of the leptin receptor (OB-Rb) shows high expression peaks in the feeding centers of the hypothalamus [21], consistent with leptin being the afferent signal informing the CNS of the body fat status. However, obese people often have elevated leptin levels with limited effects of leptin administration. This is likely due to desensitization, via the saturable transport of leptin across the blood-brain barrier and abnormalities at the level of OB-Rb activation and/or signal transduction [22].

The primary role of leptin in metabolic homeostasis is to provide to the hypothalamus the information on the amount of body fat, thereby modulating central nervous system functions that regulate food intake and energy balance [23,24]. Solely via this neuroendocrine loop, leptin was believed to control bone mass. For example, in obese children, an increase in height velocity is concomitant with acceleration of bone epiphyseal maturation of the growth plate [25] and leptin levels are increased and correlate positively with fat mass [26]. Hence, leptin was believed to be the neuroendocrine link between fat and bone mass [27-29]. Indeed, leptin increases the release of osteocalcin, an osteoblast-specific protein, via a hypothalamic relay [30]. Moreover, in fetal mice leptin increases growth of primary ossification centers [31], and leptin modulates osteogenesis [29,32,33]. Recent data also indicate that locally produced leptin may be more important than circulating leptin in regulation of bone metabolism [16,17,29], while body mass influences cortical bone mass independent of leptin signaling [34]. Leptin administration to a natural leptin knockout mouse model (ob/ob) increases bone mineral density (BMD) as well as limb length [35]. This positive effect on bone turnover may be linked to its effect on both IL-6 and the osteoprotegerin (OPG)/RANKL system [33,36]. Leptin enhances metabolic markers in osteoblasts namely alkaline phosphatase activity, osteocalcin, Coll 1 α1 chains, Insulin-like Growth factor-1 and Transforming Growth Factor-β1 (TGF-β1) levels by approximately 40% [36], parameters which we previously showed to be all increased in OA Ob compared to normal [11,12].

Leptin was found by immunohistochemistry in OA cartilage and in osteophytes, while few staining could be found in normal tissues [37], and leptin levels correlated with cartilage destruction. Moreover, differential expression of leptin and leptin receptor Ob-Rb was also recently uncovered between minimally affected and advanced OA cartilage [38]. Synovial fluid leptin levels also correlate with the severity of OA [39]. However, there are at present no key data on the presence or role of leptin in osteoblasts from the subchondral bone tissue of normal or OA individuals. Hence, this study was aimed at: i) identifying the source of leptin in OA bone tissue by measuring leptin expression and release by normal and OA Ob; ii) determining if exogenous leptin could alter cell proliferation of OA Ob; and iii) evaluating if local leptin production is responsible for abnormal production of phenotypic markers in OA Ob.

## Materials and methods

### Patients and clinical parameters

Tibial plateaus were dissected away from the remaining cartilage and trabecular bone under sterile conditions from OA patients who had undergone total knee replacement surgery as previously described [11,12,40]. A total of 64 patients (aged 71.5 ± 9.9 years) classified as having OA according to the recognized clinical criteria of the American College of Rheumatology were included in this study [41]. OA grade ranged from moderate to severe in these patients. None of the patients had received medication that would interfere with bone metabolism, including corticosteroids, for six months before surgery. A total of 16 subchondral bone specimens of tibial plateaus from normal individuals (aged 62.2 ± 18.9 years) were collected at autopsy within 12 h of death. These were used following the establishment that they had not been on any medication that could interfere with bone metabolism or had any bone metabolic disease. Individuals showing abnormal cartilage macroscopic changes and/or subchondral bone plate sclerosis were not included in the normal group. All human materials were acquired following a signed agreement by patients undergoing knee surgery or their relatives for the specimens collected at autopsy following the Centre Hospitalier de l'Université de Montréal (CHUM) ethical committee guidelines.

### Preparation of primary subchondral bone cell culture

Isolation of subchondral bone plate and the cell cultures were prepared as we recently described [42]. At confluence, cells were passaged once at 25,000 cells/cm^2 ^and grown for five days in HamF12/DMEM media (Sigma-Aldrich, Oakville, Ontario, Canada) containing 10% FBS before specific assays. These cells were incubated with the same media containing 0.5% FBS. After 24 hours of preconditioning, cells were incubated for either an additional 48 hours in HamF12/DMEM media containing 0.5% FBS and the indicated treatments for the determination of phenotypic markers, or they were incubated for an additional 24 hours in the same media in presence or absence of increasing doses of leptin and the indicated treatments for the determination of the expression of leption or OB-Rb, or they were incubated for 15 minutes with increasing doses of leptin in preparation for Western blot analysis of p42/44 and p38. For the determiniation of phenotypic markers, cells were either treated with 1 μg/ml recombinant human leptin (rhleptin, Calbiochem, San Diego, California, USA), 10 μg/ml recombinant human leptin R/Fc chimera (R&D Systems, Minneapolis, MN, USA) that neutralizes the activity of rhleptin, 100 μM Tyrphostin (AG490, Sigma-Aldrich), 75 μM piceatannol (Pce, Sigma-Aldrich), or the vehicle. Supernatants were collected at the end of the incubation and kept at -80°C prior to assays. Cells were either prepared for SDS-PAGE separation or RT-PCR experiments. Cells prepared for SDS-PAGE separation were lysed with RIPA buffer (50 mM Tris HCl pH 7.4, 1% NP-40, 0.5% Na-deoxicholate, 0.1% SDS, 150 mM NaCl with the following inhibitors: 10 μg/ml aprotinin, 10 μg/ml leupeptin, 10 μg/ml pepstatin, 10 μg/ml O-phenatroline, 1 mM Na-orthovanadate, 1 mM DTT), and kept at -80°C prior to assays. Protein determination was performed by the bicinchoninic acid method [43].

### Phenotypic characterization of human subchondral Ob cell cultures

Phenotypic features of Ob were determined by evaluating 1,25(OH)_2_D_3_-dependent (50 nM) alkaline phosphatase activity and osteocalcin release, and by measuring the release of the carboxy-terminal propeptide of collagen type 1 (CICP) in cells treated or not for their last 48 hours of culture with recombinant human leptin R/Fc chimera to neutralize the activity of leptin, 100 μM tyrphostin (AG490) or 75 μM piceatannol (Pce), inhibitors of leptin signaling, or with siRNA directed against leptin or OB-Rb (see below). Alkaline phosphatase activity was determined on cell aliquots by substrate hydrolysis using p-nitrophenylphosphate (PNPP), and osteocalcin release was determined in cell supernatants using an EIA as previously described [11,12]. CICP was determined using a selective ELISA (Quidel Corporation, Cedarlane, Hornby, Ontario, Canada) in conditioned media from confluent OA Ob incubated in HAMF12/DMEM media containing 0.5% bovine serum albumine (BSA). CICP release was then reported as ng per cellular proteins. Transforming growth factor-β1 (TGF-β1) was measured in supernatants using a highly specific Quantikine ELISA assay from R&D Systems (Minneapolis, MN, USA). The sensitivity of the assay is 7 pg/ml and is a very specific assay that does not cross react with related cytokines/growth factors when tested at saturating concentrations. Cellular proliferation was assessed using two complementary approaches: the BrdU cell proliferation assay as described in the system's manual from Calbiochem (San Diego, California, USA) and MTT assay as described by Zhao *et al*[44]. Cells were plated at 10,000 cells/cm^2 ^in 96-well plates in Ham F12/DMEM media containing 10% FBS. After overnight attachment, cells were fed Ham F12/DMEM media containing 0.5% FBS for 24 hours prior to stimulation with or without increasing doses of recombinant human leptin as indicated for another 24 hours of incubation.

### RT-PCR assays

For RT-PCR assays, total cellular RNA from normal and OA Ob was extracted with the TRIzol™ reagent (Invitrogen, Burlington, Ontario, Canada) according to the manufacturer's specifications and treated with the RNA-free™ Dnase Treatment and Removal kit (Ambion, Austin, TX, USA) to ensure complete removal of chromosomal DNA. The RNA was quantitated using the RiboGreen RNA quantification kit (Molecular Probes, Eugene, OR, USA). The RT reactions were primed with random hexamers with 1 μg of total RNA in a 100 μl final reaction volume followed by PCR amplification as previously described [40] using 20 pmol of each specific PCR primers (see below). The amplification of all mRNA species was performed separately from GAPDH mRNA amplification to avoid substrate depletion. After amplification, DNA was analyzed on an agarose gel and visualized by ultraviolet detection.

Real-time quantification of leptin and GAPDH mRNA was performed in the GeneAmp 5700 Sequence Detection System (Applied Biosystems, Foster City, CA, USA) with the 2× Quantitect SYBR Green PCR Master Mix (Qiagen, Missisauga, Ontario, Canada) used according to the manufacturer's specifications. Primers used were: 5'-GGCTTTGGCCCTATCTTTTC-3' (sense) and 5'-GGATAAGGTCAGGATGGGGT-3' (antisense) for Lep1; 5'-CCTCATCAAGACAATTGTCACC-3' (sense) and 5'-CAGCATGTCCTGCAGAGACC-3' (antisense) for Lep2; 5'-GCCAGAGACAACCCTTTGTTAAA-3' (sense) and 5'-TGGAGAACTCTGATGTCCGTGAA-3' (antisense) for OB-Rb; 5'-CAGAACATCATCCCTGCCTCT-3' (sense) and 5'-GCTTGACAAAGTGGTCGTTGAG-3' (antisense) for GAPDH. Amplicons were 197, 376, 417 and 319 bp, respectively. In brief, 100 ng of the cDNA obtained from the RT reactions were amplified in a total volume of 50 μl consisting of 1× Master mix, uracil-N-glycosylase (UNG, 0.5 Unit, Epicentre Technologies, Madison, WI, USA) and the gene-specific primers which were added at a final concentration of 200 nM. The tubes were first incubated for two minutes at 50°C (UNG reaction), then at 95°C for 15 minutes (UNG inactivation and polymerase activation) followed by 40 cycles consisting each of denaturation (94°C for 15 seconds), annealing (60°C for 30 seconds), extension (72°C for 30 seconds) and data acquisition (77°C for 15 seconds) steps. The data were collected and processed with the GeneAmp 5700 SDS software and given as threshold cycles (Ct), corresponding to the PCR cycle at which an increase in reporter fluorescence above baseline signal can first be detected. When comparing normal and OA basal expression levels, the Ct were converted to the number of molecules and the values for each sample calculated as the ratio of the number of molecules of the target gene/number of molecules of GAPDH.

### Inhibition of leptin and OB-Rb expression using siRNA

We used a siRNA technique to transiently inhibit leptin or OB-Rb expression in OA Ob. SiRNA were obtained from Dharmacon (Lafayette, CO, USA) and we followed the manufacturer's directions for their preparation. Briefly, OA Ob were split at 100,000 cells/ml. Leptin or OB-Rb siRNA (a set of four different siRNA per gene) or scramble RNA (basal condition) was added to OA Ob at a final concentration of 100 ng/ml with 6 μl Hi-perfect (Quiagen, Missisauga, ON, Canada) per 100 μl total volume in BGJb media without serum for one hour on Day 0 and Day 3. Cells were then fed BGJb media with 10% FBS containing 50 nM 1,25(OH)_2_D_3 _until Day 7, with media changes every two days. Cells were harvested in either ALPase buffer to perform ALP and protein determination or in TRIzol to prepare for RT-PCR to detect changes in leptin and OB-Rb levels. Supernatants were kept for the determination of osteocalcin.

### Western immunoblotting

The cell extracts were loaded on polyacrylamide gels and separated by sodium dodecyl sulfate-polyacrylamide gel electrophoresis (SDS-PAGE) under reducing condition [45]. Loading of the protein was adjusted according to the cellular protein concentration of each specimen. The proteins were then electrophoretically transferred onto Polyvinylidene Fluoride (PVDF) membranes (Boehringer Mannheim, Penzberg, Germany), and immunoblotting was performed as described in the ECL Plus Western blotting detection system's manual (Amersham Pharmacia Biotech, Piscataway, NJ, USA). Rabbit anti-leptin receptor at a dilution of 1:1,000 (Cedarlane, Hornby, Ontario, Canada), rabbit anti-human actin at a dilution of 1:10,000 (Sigma-Aldrich), rabbit anti p42/44 at a dilution of 1:5,000 (Cell Signaling Technology, Beverly, MA, USA), rabbit anti-phosphorylated p42/44 (Thr202/Tyr204) at a dilution of 1:5,000 (Cell Signaling Technology), rabbit anti p38 at a dilution of 1:2,000 (Cell Signaling Technology), and anti-phosphorylated p38 at a dilution of 1:1,000 (Cell Signaling Technology) as primary antibodies, and goat anti-rabbit IgG as secondary antibodies at a dilution of 1:20,000 (Upstate Biotechnology, Lake Placid, NY, USA) were used for the assays.

Densitometry analysis of western blot films was performed on a Macintosh Mac OS 9.1 computer using the public domain NIH Image program developed at the U.S. National Institutes of Health with the Scion Image 1.63 program [46].

### Evaluation of leptin production

Leptin was evaluated in Ob-conditioned media. Confluent Ob were cultured for 48 h in HAMF12/DMEM media containing 0.5% FBS. At the end of the incubation, their conditioned-media were concentrated five-fold using Amicon Ultra-4 filters (Ultracil-10 k, Millipore Corporation, Bedford, MA, USA) with a cutoff of 10 kDa. Samples were centrifuged at 1,000 g for 15 minutes at 4°C. The concentrated conditioned media were then tested for leptin using a selective high sensitivity ELISA (R&D Systems). The sensitivity of the assay was 7.8 pg/ml and the intra-assay precision is 3.2 ± 0.2%.

### Statistical analysis

All quantitative data are expressed as mean ± SEM. Statistical analysis was performed by an ANOVA analysis of variance for dose-response experiments, followed by adequate subtests when statistical significance was reached. A non parametric Mann-Whitney U statistical test was performed for all other experiments and *P *values < 0.05 were considered statistically significant.

## Results

### Expression and production of leptin in osteoblasts

**Figure 1:**
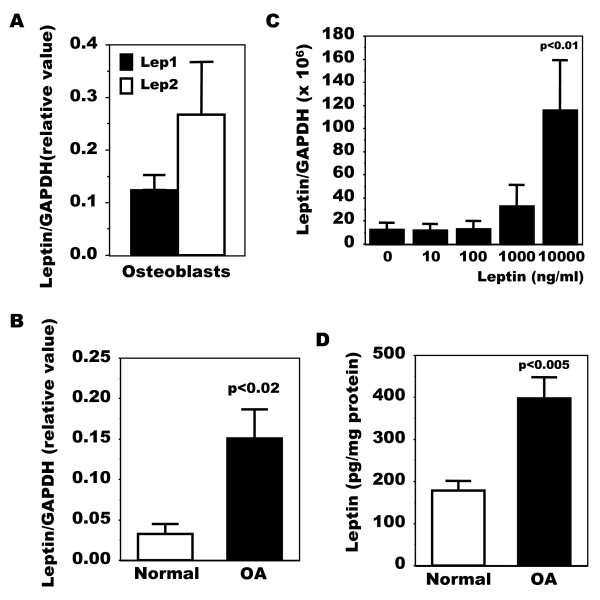
**Production of leptin in normal and OA osteoblasts**. The expression of leptin was first determined by qPCR. Confluent osteoblasts (Ob) were lized in TRIzol and RNA extracted as described in Material and methods. RNA (1 μg) was reversed transcribed followed by qPCR amplification of 100 ng cDNA using specific primers for leptin and GAPDH. The data were processed with the GeneAmp 5700 SDS software and given as threshold cycle (Ct), corresponding to the PCR cycle at which an increase in reporter fluorescence above baseline signal can first be detected. The Ct was converted to the number of molecules and the values for each sample calculated as the ratio of the number of molecules of the target gene/number of molecules of GAPDH. **A**) Quantification of leptin mRNA using Lep1 and Lep2 primers. Results are given as the mean value of markers relative to GAPDH ± SEM of n = 4 OA preparations. **B**) Quantification of leptin mRNA levels in normal and OA Ob using Lep1 primers. Results are the mean ± SEM of n = 5 normal and n = 15 OA individual Ob preparations. **C**) OA Ob were exposed to increasing doses of leptin and lepin mRNA levels were determined using Lep1 primers. Results are the mean ± SEM of n = 4 preparations. The protein production of leptin was next detected using a very selective ELISA. Conditioned-media of confluent normal and OA Ob incubated in HAM's F12/DMEM media containing 0.5% FBS for their last 48 hours of culture were recuperated and stored at -80°C. **D**) Aliquots were taken to measure leptin using a very sensitive ELISA. Results are the mean ± SEM of n = 5 normal and n = 6 OA individual Ob preparations.

**Figure 2:**
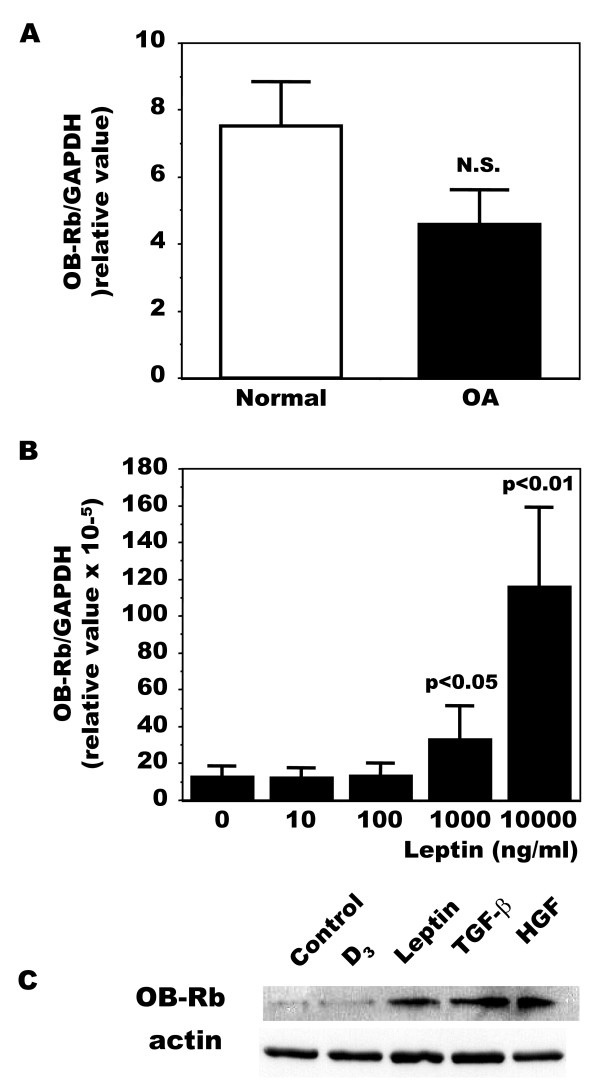
**Production of leptin receptors (OB-Rb) in normal and OA osteoblasts**. The expression of leptin receptors was first determined by qPCR. **A**) Confluent Ob were lized in TRIzol and RNA extracted as described in Material and Methods. RNA was reversed transcribed followed by PCR amplification of 100 ng cDNA as described in Figure 1 using OB-Rb and GAPDH primers. Results are the mean ± SEM of n = 7 normal and n = 19 OA Ob preparations, *P *< 0.004 vs normal and OA. **B**) OA Ob were incubated for 24 hours with increasing concentrations of exogenous leptin. Cells were then lyzed and used for PCR amplification of OB-Rb as in A. Results are the mean ± SEM of n = 6 OA Ob preparations. Second, the production of leptin receptors was determined by Western blot analysis. **C**) Confluent Ob were treated for 48 hours with or without 1,25(OH)_2_D_3 _(50 nM), leptin (100 ng/ml), TGF-β1 (10 ng/ml) or HGF (10 ng/ml). The cells were then lized in RIPA buffer prior to separation using SDS-PAGE and Western blotting using specific antibodies to OB-Rb.

We first questioned if human OA osteoblasts (Ob) expressed leptin compared to normal Ob using real-time RT-PCR with two different set of primers, one described by Dumond *et al*[37] for rat samples and adapted to the human sequence, and the other by Gordeladze *et al*[16] for primary human osteoblasts. Using both sets of primers we detected leptin expression in OA Ob (Figure 1A). We next evaluated if OA Ob produced variable levels compared to normal Ob. Using real-time RT-PCR we observed that OA Ob produced about approximately five-fold more leptin mRNA than normal Ob using one set of primers (Figure 1B). Since leptin has been shown to promote its own expression [47], we next determined if this could be the case in OA Ob. Indeed, leptin dose-dependently stimulated its own expression (Figure 1C), yet this was also the case for OB-Rb expression (Figure 2B). As Ob expressed leptin, we next evaluated the capacity of Ob to synthesize leptin. As shown in Figure 1D, OA Ob released about approximately two-fold more leptin than normal Ob under basal condition when measured using a very selective ELISA.

### Expression and production of leptin receptors in osteoblasts

In order to determine if OA Ob could respond to leptin, we next evaluated the presence of the long, signaling competent, form of the leptin receptor (OB-Rb). As shown in Figure 2A using real-time RT-PCR, OA Ob expressed slightly less OB-Rb than normal Ob although this did not reach significance. Exogenous leptin at high concentrations significantly stimulated OB-Rb expression in OA Ob (Figure 2B). In addition, OB-Rb mRNA levels were increased by both TGF-β1 and HGF in OA Ob (not illustrated), and this increased expression was reflected at the protein level by Western blot analysis (Figure 2C). Similar Western blot results were obtained with OA chondrocytes (not illustrated).

### Role of leptin in abnormal phenotypic features of ostearthritic osteoblasts

**Figure 3:**
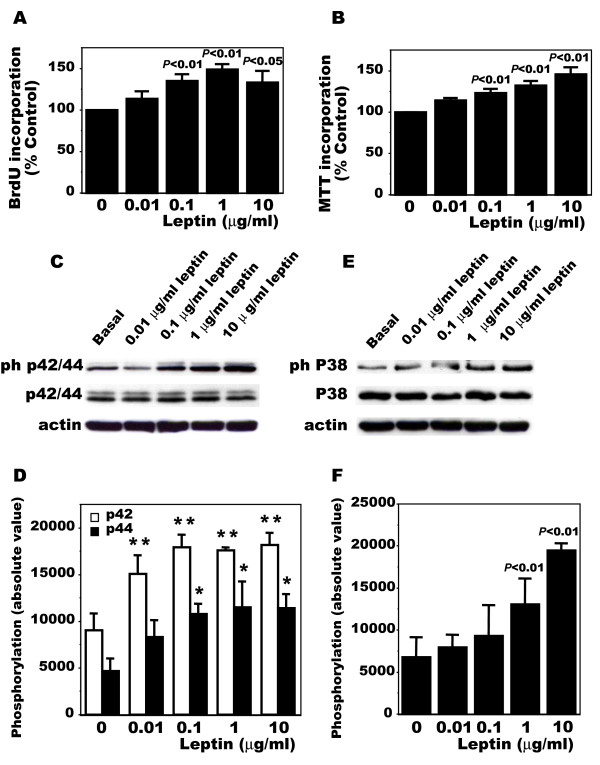
**Cellular proliferation and intracellular signaling of OA osteoblasts in response to leptin**. OA osteoblasts were plated at 10,000 cells/cm^2 ^and allowed to attach overnight in HAM's F12/DMEM media containing 10% FBS. Cells were then treated with the same media with 0.5% FBS for 24 hours prior to receiving increasing doses of leptin (10 ng/ml, 100 ng/ml, 1 mg/ml or 10 mg/ml) or the vehicle in the same media for another incubation of 24 hours. Cell proliferation was assessed by the incorporation of BrdU or MTT assay. **A**) Incorporation of BrdU by OA Ob in response to leptin; **B**) Proliferation of OA Ob by MTT assay; **C) **Representative phospho p42/44 Western blot analysis in response to increasing doses of leptin in OA Ob. **D**) Determination of phospho p42/44 levels using the NIH Image program developed at the U.S. National Institutes of Health with the Scion Image 1.63 program [46]. **E) **Representative phospho p38 Western blot analysis in response to increasing doses of leptin in OA Ob. **F) **Determination of phospho p38 levels using the NIH Image program developed at the U.S. National Institutes of Health with the Scion Image 1.63 program [46]. Values are the mean ± SEM of at least four separate experiments; *P < 0.05, **P < 0.01.

Since OA Ob expressed both leptin and leptin receptors, we tested if these cells could respond to exogenous leptin and we first determined the effect of leptin on cell proliferation. Figure 3A and 3B show that leptin dose-dependently (1 ng/ml to 10 μg/ml) stimulated cell proliferation and this effect plateaued at 100 ng/ml leptin when assessing proliferation using BrdU incorporation or MTT assay respectively. We next evaluated if the effect of leptin on cell proliferation was via the Erk 1/2 MAPK pathway as we previously showed with insulin-like growth factor 1 [45]. Indeed, in response to exogenous leptin, phospho p42/44 MAPK levels rose (Figure 3C). This effect was again dose-dependent and also plateaued around 100 ng/ml (Figure 3D). In addition, we evaluated the role of leptin on the p38 pathway. Again, leptin dose-dependently stimulated phospho p38 levels (Figure 3E) and this effect was significant at doses as low as 1 μg/ml (Figure 3F).

**Figure 4:**
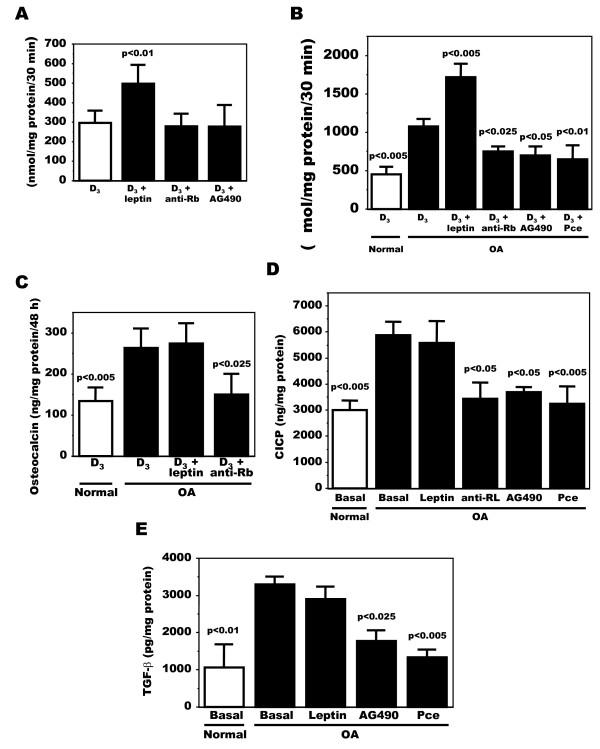
**Modulation of alkaline phosphatase, osteocalcin and collagen type 1 in OA Ob by inactivating leptin signaling**. Confluent normal and OA Ob were treated for their last two days of culture with either media alone containing 0.5% FBS with or without 1,25(OH)_2_D_3 _(50 nM) as per indicated for the individual markers. Cells were treated with either exogenous leptin, antibodies against leptin, tyrphostin (AG490, 100 μM) or Piceatannol (Pce, 75 μM) for 30 minutes prior to the addition of 1,25(OH)_2_D_3 _except for CICP that was performed in the absence of 1,25(OH)_2_D_3_. At the end of the 48 h incubation, the supernatant was kept for osteocalcin and for collagen production, and cells were lyzed in ALPase buffer prior to measuring alkaline phosphatase activity by substrate hydrolysis. **A**) Results of alkaline phosphatase activity for normal OB; **B**) Results of alkaline phosphatase activity for OA OB; **C**) Results of osteocalcin release by OA Ob; **D**) Results of CICP production; **E**) Confluent OA Ob were incubated in Ham's F12/DMEM media without serum and containing 1% ITS. Cells were treated with or without exogenous leptin, tyrphostin (AG490, 100 μM) or Piceatannol (Pce, 75 μM) for their last 48 hours of culture. Results of TGF-β1 levels in supernatants are shown. The results are the mean ± SEM of n = 4 normal and n = 9 OA Ob preparations.

Leptin influences the synthesis of phenotypic markers and inflammatory mediators in a number of cells and in particular can increase phenotypic markers in primary human Ob [36]. Because OA Ob responded to exogenous leptin, we then questioned if the endogenous elevated leptin production observed in OA Ob could be responsible for the abnormal phenotypic markers of these cells. Hence, we measured alkaline phosphatase activity, osteocalcin release and the production of CICP under basal condition and in the presence leptin or of a recombinant human leptin R/Fc chimera (anti-Rb) that neutralizes the activity of leptin. First, in preliminary assays we tested if exogenous leptin, the recombinant leptin R/Fc chimera or the antagonist of leptin signaling AG490 would alter alkaline phosphatase activity in normal Ob. Indeed, leptin addition to normal Ob stimulated vitamin D_3_-dependent alkaline phosphatase activity, however, neither anti-Rb nor AG490 had any effect on this activity (Figure 4A), indicating no cytotoxic effects of these treatments on normal Ob. Hence, we next tested their effect on OA Ob and compared it to basal levels of these phenotypic markers in normal Ob run in parallel. Here, the inhibition of leptin signaling in OA Ob in response to 100 μM AG490 or 75 μM Pce, selective inhibitors of leptin intracellular signaling, reduced ALPase and CICP to values similar to normal Ob (Figure 4B and 4D), whereas the effect of these inhibitors on osteocalcin secretion could not be tested since they interfered with the EIA method. These inhibitors did not promote any significant cell death as assessed by total protein content and cell count by trypan blue exclusion (not illustrated). In addition, anti-Rb inhibited all these activities in OA Ob (Figure 4B to 4D). We then questioned if exogenous leptin could promote these activities. The addition of exogenous leptin to OA Ob enhanced vitamin D_3_-dependent alkaline phosphatase activity (Figure 4B) as in normal Ob (Figure 4A), but it failed to further stimulate osteocalcin release (Figure 4B) or collagen type 1 production (Figure 4C) above their already elevated values in OA Ob.

As another key feature of OA Ob that distinguishes them from normal Ob is their enhanced production of TGF-β1 [12], and because leptin has been shown to stimulate TGF-β1 synthesis in other cells, we evaluated if high levels of TGF-β1 in OA Ob could be due to a response to endogenous leptin via a paracrine/autocrine stimulation. As shown in Figure 4E, TGF-β1 levels in OA Ob were elevated compared to normal Ob and the presence of AG490 or Pce reduced by approximately 50% and approximately 60% the endogenous levels of TGF-β1 in OA Ob, reducing them to near normal values.

**Figure 5:**
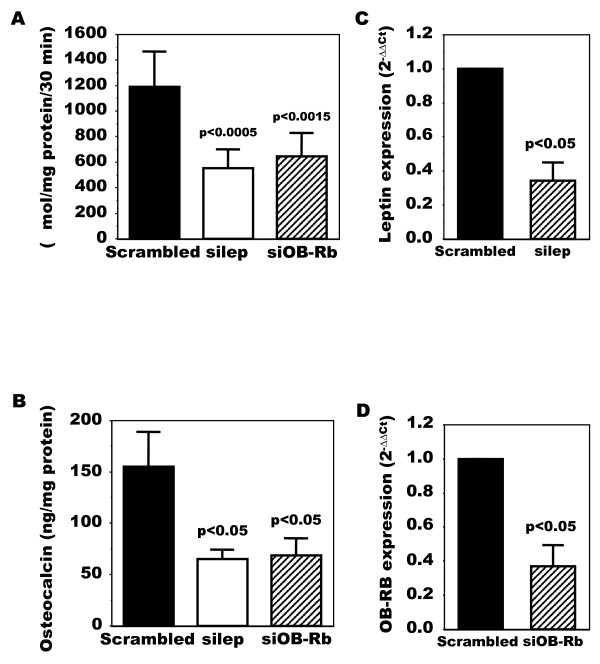
**Modulation of alkaline phosphatase and osteocalcin release in OA Ob by inactivating leptin or leptin signaling**. OA Ob were treated with siRNA for either leptin or OB-Rb or a scrambled RNA as described in Material and methods. Cells were then used to determine alkaline phosphatase activity and osteocalcin release. **A**) Results of alkaline phosphatase activity in response to leptin or OB-Rb siRNA treatments. **B**) Results of osteocalcin release in response to leptin or OB-Rb siRNA treatments. **C**) Leptin expression in response to siRNA. **D**) OB-Rb expression in response to siRNA. Results are the mean ± SEM of n = 6 OA Ob preparations.

Last, using siRNA techniques, we next evaluated if inhibiting leptin or OB-Rb would abrogate the response of OA Ob to endogenous leptin production. Indeed, as shown in Figure 5A, siRNA against leptin reduced alkaline phosphatase activity about 60% compared to a scrambled RNA. A similar observation could be made for osteocalcin (Figure 5B). Likewise, inhibiting OB-Rb expression using siRNA techniques also reduced ALP and OC about 60% in OA Ob (Figure 5A and 5B). Figures 5C and 5D show that specific siRNA inhibition reduced leptin and OB-Rb expression 60 and 55% respectively in these cells compared to a scrambled RNA.

## Discussion

In recent years, a key role of leptin in OA has been proposed, primarily based on the observation that human articular cartilage from OA patients showed elevated leptin levels using immunohistochemistry [37,38]. Leptin was previously known to be present in chondrocytes of the growth plate in young animals [27] and in fetal mice [39] yet this information was lacking in adult cartilage until Dumond *et al*[37] and Simopoulou *et al* described differential expression of both leptin and leptin receptors between normal and OA cartilage [38]. In the present study, we show that subchondral osteoblasts also have high levels of expression for leptin. Together with the study by Simopoulou *et al*[38], this could suggest that the presence of leptin in articular cartilage could also be due, at least in part, to its local production in subchondral bone tissue. Indeed, since OA Ob expressed more leptin mRNA and produced more leptin, this could explain the higher protein levels found in OA cartilage compared to normal tissue [37] if leptin can seep to the articular cartilage via either the enhanced micro-circulation present in the subchondral bone plate and the deep layer of the articular cartilage or via microcracks [48,49].

Our study also provided contrasting data on the regulation of leptin expression by OA Ob. We first tried to demonstrate a possible link between elevated TGF-β1 and HGF levels produced by OA Ob [12,50]. and the expression of leptin by these cells. As previously showed for a number of tissues [51], TGF-β1 reduced significantly the expression of leptin mRNA by OA Ob (not illustrated). In contrast, HGF was without any significant effect on leptin expression. Second, 1,25(OH)_2_D_3 _stimulated several fold leptin expression by OA Ob. This is in sharp contrast to available data with adipocytes [52] that show a powerful inhibition of leptin expression in response to 1,25(OH)_2_D_3_. Hence, adipocytes and Ob behave differently and this could be a crucial step in OA Ob. Indeed, OA Ob show enhanced responses to 1,25(OH)_2_D_3 _stimulation [11,40], and this could possibly contribute to the enhanced expression of leptin.

Inasmuch as leptin expression is enhanced and its endogenous production is elevated in OA Ob, this could explain the slight reduction in OB-Rb expression in these cells compared to normal due to the continuous exposure to leptin. This reduction in OB-Rb expression was also paralleled by a slight decrease of leptin receptors at the protein level as detected by Western blot analysis. However, OA Ob could still respond to an acute exogenous leptin stimulation with an increase in OB-Rb expression. In addition, OB-Rb production was stimulated by TGF-β1 and HGF in OA Ob, and this would indicate that the receptors could still be regulated normally in these cells. Nonetheless, this reduction in OB-Rb does not lead to a reduction of the response of OA Ob to leptin as was demonstrated here. Indeed, OA Ob responded to exogenous addition of leptin with an increase in cell proliferation, phospho p42/44 MAPK, and in alkaline phosphatase activity as was previously reported for primary human osteoblasts [36]. In addition, leptin also stimulated phospho p38 levels in OA Ob in the present study downstream to a stimulation of the JAK2/STAT3 pathway (not illustrated). A previous study indicated a JAK/STAT dependent involvement of the p42/44 MAPK and p38 kinase pathways in the chondrogenic ATDC5 cell line in response to leptin stimulation [53]. In contrast, leptin could not increase osteocalcin secretion nor CICP or TGF-β1 levels in OA Ob. This could either indicate that alkaline phosphatase is more sensitive to leptin stimulation than the other markers or else that osteocalcin, CICP and TGF-β1 production are less sensitive to leptin in these cells. Conversely, blocking OB-Rb signaling with inactivating antibodies reduced the production of alkaline phosphatase, osteocalcin, and CICP in OA Ob. This is a key observation since OA Ob show abnormal phenotypic features, namely elevated alkaline phosphatase activity, osteocalcin release, collagen type 1, IGF-1 and TGF-β1 production [11,12,40,54], all features that can be increased in response to leptin [36]. Last, tyrphostin and piceatannol, selective inhibitors respectively of the JAK2/STAT3 and JAK1/STAT3 pathways involved in leptin signaling [55], reduced the activity of alkaline phosphatase, and the production of collagen type 1 and TGF-β1 by OA Ob. Thus, these data suggest first, that the abnormal features of OA Ob could be related to their endogenous elevated production of leptin, and second, that this response to leptin involves the JAK2/STAT3 downstream MAPK targets, Erk1/2 and p38. Last, we showed a reduction in alkaline phosphatase activity and osteocalcin release by silencing leptin with siRNA or silencing OB-Rb with siRNA in OA Ob. This again is pointing toward a key role of endogenous leptin to regulate these activities in OA Ob.

Inasmuch as leptin contributes to stimulate the production of IGF-1 and TGF-β1 by human osteoblasts [36], our data would also indicate that leptin is a key signal in OA pathophysiology. Indeed, both growth factors have been implicated in the initiation and/or progression of OA and we previously showed that both growth factors were elevated in *in vitro *subchondral osteoblasts isolated from OA patients [12,56] Moreover, leptin can alter the signaling of IGF-1 in a number of cell systems [57-59] and we previously reported that IGF-1 signaling is abnormal in OA Ob [45]. We also previously showed that HGF is not produced by chondrocytes but is produced in higher abundance by OA Ob [50] whereas HGF can increase OB-Rb levels in chondrocytes (not illustrated). Hence, the presence of elevated levels of leptin [37,38] in OA cartilage, and of elevated expression of OB-Rb [38,60] possibly due to elevated HGF levels [50] derived from osteoblasts of the subchondral bone plate, could therefore promote the response to leptin in OA chondrocytes.

The failure of leptin to stimulate all markers of osteoblasts whereas inhibiting leptin signaling modified all parameters is puzzling. However, an abnormal response to leptin in OA Ob is likely and resembles what we previously observed for the response to IGF-1. First, an increase in phospho-PTP1B/Syp may be crucial for leptin signaling as PTP1B/Syp controls STAT3 phosphorylation and its interaction with target genes, a key signaling pathway for leptin [61]. Since increased hypothalamic PTP1B levels can contribute to leptin resistance [62], our previous demonstration that PTP1B/Syp levels and phosphorylation are increased in OA Ob [45] suggests that OA Ob could actually be resistant to some, yet not all, leptin signaling since leptin did increase the phosphorylation of Erk1/2 and p38 in OA Ob (our present data). Second, leptin promotes tyrosine phosphorylation of SHC proteins and the association of SHC with Grb2 in human embryonic cells HEK 293 [63], whereas we previously showed that Grb2 interaction with IRS-1 and SHC is abnormal in OA Ob [45]. Third, leptin can modulate its effects on human Ob via an inhibition of apoptosis [36], and we observed a reduction in Bax-α to Bcl2 expression in OA Ob compared to normal Ob [45] an indication of reduced apoptosis. Indeed, leptin increases the proliferation of human osteoblast-like SaOS-2 cells via Erk1/2 [64], and we also showed that OA Ob have an enhanced proliferation rate and increased phospho Erk 1/2 levels in response to leptin. Last, we showed that leptin stimulates alkaline phosphatase activity in OA Ob yet failed to stimulate any further osteocalcin release, collagen production or TGF-β1 levels above their already elevated levels compared to normal Ob, whereas inhibiting leptin signaling clearly showed an inhibition of all these parameters. Previous studies have shown that long term exposures to leptin are needed to modify the expression of collagen type 1, TGF-β1 and osteocalcin [36] whereas the inhibition of collagen synthesis with the JAK inhibitor AG490 (tyrphostin) is rapid [65] similar to what we observed here. Taken together, these data would suggest that a number of, yet not all, signaling pathways are altered in OA Ob in response to leptin. Conversely, partners of leptin signaling may be abnormal in OA Ob and this may stem from the possible abnormal cross-talk between the IGF-1 signaling, already abnormal in OA Ob [45], and leptin signaling pathways in these cells. These hypotheses remain to be explored.

## Conclusions

This study indicates that OA Ob expressed and released more leptin than normal. This elevated production of leptin is responsible, at least in part, for the abnormally elevated levels of biomarkers of OA Ob compared to normal, and to elevated production of TGF-β1. Together, these data indicate a key role of leptin in OA pathophysiology.

## Abbreviations

ALP: alkaline phosphatise; BMD: bone mineral density; BMI: body mass index; BSA: bovive serum albumin; CICP: collagen type 1 carboxyl terminal peptide; CNS: central nervous system; IGF-1: insulin-like growth factor 1; MTT: (3-(4,5-Dimethylthiazol-2-yl)-2,5-diphenyltetrazolium bromide; OA: osteoarthritis; Ob: osteoblasts; OB-Rb: leptin receptor long form; OC: osteocalcin; OPG: osteoprotegerin; PCR: polymerase chain reaction; PNPP: para-nitrophenyl phosphate; PVDF: Polyvinylidene Fluoride; TGF-β1: transforming growth factor beta-1.

## Competing interests

The authors declare that they have no competing interests.

## Authors' contributions

MSM and MAA performed the experiments, participated in the statistical analysis and the interpretation of data, and drafted the manuscript. AD performed the experiments, participated in the statistical analysis and interpretation of data, and reviewed the manuscript. ML participated in the recruitment of OA patients, collection of samples, interpretation of data, and reviewed the manuscirpt. DL participated in the design of the study, performed the statistical analysis and the interpretation of data, and drafted the manuscript. All authors read and approved the final manuscript.
